# Genomic analysis of vB_PaS-HSN4 bacteriophage and its antibacterial activity (in vivo and in vitro) against *Pseudomonas aeruginosa* isolated from burn

**DOI:** 10.1038/s41598-023-50916-5

**Published:** 2024-01-23

**Authors:** Solmaz Rafiei, Majid Bouzari

**Affiliations:** Department of Cell and Molecular Biology and Microbiology, Faculty of Biological Science and Technology, University of Isfahan, Hezar-Jereeb Street, Isfahan, 81746-73441 Iran

**Keywords:** Bacteriology, Virology, Phage biology, Antimicrobial resistance

## Abstract

The most frequent infections caused by *Pseudomonas aeruginosa* are local infections in soft tissues, including burns. Today, phage use is considered a suitable alternative to cure infections caused by multi-drug-resistant (MDR) and extensively drug-resistant (XDR) bacteria. We investigated the potential of a novel phage (vB_PaS-HSN4) belonging to *Caudoviricetes* class, against XDR and MDR *P. aeruginosa* strains in vivo and in vitro. Its biological and genetic characteristics were investigated. The phage burst size and latent were 119 and 20 min, respectively. It could tolerate a broad range of salt concentrations, pH values, and temperatures. The combination with ciprofloxacin significantly enhanced biofilm removal after 24 h. The genome was dsDNA with a size of 44,534 bp and encoded 61 ORFs with 3 tRNA and 5 promoters. No virulence factor was observed in the phage genome. In the in vivo infection model, treatment with vB_PaS-HSN4 increased *Galleria mellonella* larvae survival (80%, 66%, and 60%) (MOI 100) and (60%, 40%, and 26%) (MOI 1) in the pre-treatment, co-treatment, and post-treatment experiments, respectively. Based on these characteristics, it can be considered for the cure of infections of burns caused by *P. aeruginosa.*

## Introduction

A significant source of hospital-acquired infections is attributed to *P. aeruginosa*, particularly among patients with burns, compromised immune systems, and cystic fibrosis^1^. This bacterium is the fourth main cause of hospital-acquired infections after *Staphylococcus aureus, Klebsiella pneumonia,* and *Escherichia coli*^2^*. Pseudomonas* is a genus in the *Pseudomonadaceae* family and the most common species of it in human infections is *P. aeruginosa*, which is a Gram-negative, spore-free, and motile bacillus. The ability of this bacterium to produce many pathogenic compounds such as alginate, proteases, pyocyanin, phospholipase C, and colonization of the host cells and biofilm formation makes it one of the most important pathogens^3–5^. It causes pneumonia, postoperative, urinary tract, burns, and ear infections, bacteremia, and endocarditis. The mortality rate in immunocompromised patients with nosocomial pneumonia caused by *P. aeruginosa* is approximately 70% and sepsis caused by this bacterium is a serious complication following burn infection^6,7^. The ubiquitous spread of *P. aeruginosa* in the environment has drawn attention to this bacterium. Minimal nutritional requirements and high resistance to antibiotics allow the bacterium to survive in a variety of hosts^5^. The dissemination of extensively drug-resistant (XDR) and multi-drug resistant (MDR) *P. aeruginosa* strains due to inherent and acquired resistance, including horizontal transfer of resistance genes and genetic changes caused by mutation, has caused antibiotic treatment of *Pseudomonas* infections to fail^8,9^. For this, investigators are searching for new treatment strategies. Bacteriophages, because of their high specificity for their host, low environmental impact, ability to penetrate biofilm, and other advantages are suitable candidates for treatment^10,11^. The first bacteriophage against *P. aeruginosa* was isolated in the middle of the 20th century^12^. McVay et al.^13^, used a cocktail of phages consisting of three ATCC 14205-B1, ATCC 12175-B1, and ATCC 14203-B1 phages to treat burn infection caused by *P. aeruginosa*. This cocktail contained 1 × 10^8^ PFU of each mentioned phage. The results showed that this phage cocktail dramatically reduces the mortality in the treated mice, so that the percentage of live mice from 6% in the control group reached 22–87% in the treated group. Jault et al.^14^ succeeded in creating a cocktail of phages consisting of 12 phages from the *Podoviridae* and *Myoviridae* families called PhagoBurn. The remedial effect of this cocktail on patients with burn infection caused by *P. aeruginosa* was investigated. Compare to the standard of care, the bacterial count was decreased and less serious adverse effects were observed in phage cocktail-treated individuals. Marashi et al.^15^ isolated PaBa1, PaBa2, and PaBa3 phages from the *Myoviridae* and *Podoviridae* families against XDR strains of *P. aeruginosa*, causing burn infections. Phage PaBa1 and the phage cocktail prepared from the three mentioned phages were able to lyse 62.5% and 67.5% of *P. aeruginosa* strains, respectively. The results indicated that the isolated phages are potent to treat and prevent infections caused by this bacterium. Adnan et al.^16^ isolated phage MA-1 against multidrug-resistant strains of *P. aeruginosa*. This phage belonged to the *Myoviridae* family. The results showed that MA-1 phage was significantly able to remove planktonic cells and biofilm caused by *P. aeruginosa*. The objective of the present study was to isolate a new lytic phage against *P. aeruginosa* and to investigate and analyze its biological, genomic, and proteomic characteristics.

## Results

### *P. aeruginosa* isolation

In this study, a total of 100 patients with the burn infection were tested and colonies of different bacteria presumably *Staphylococcus*, *Klebsiella*, *Acinetobacter* and *Pseudomonas* were detected in selective media. Circular, mucoid, and smooth colonies with a grape-like odor were primarily considered as *P. aeruginosa*. These isolates (90) also exhibited β-hemolysis on blood agar and were able to grow on MacConkey agar, and did not ferment lactose. Furthermore, on Muller-Hinton agar, the isolates produced a distinctive pigment. The color of this pigment ranged from bluish-green to yellowish-green. In the biochemical tests, the motile bacteria being Gram-negative, catalase, oxidase, and citrate positive, and urease, Indole, H2S and MR-VP negative, TSI (Alk/Alk), glucose non-fermenting, grown on cetrimide agar and grown at 42 °C were considered as *P. aeruginosa*. PCR results confirmed all the isolates (90 isolates) diagnosed by biochemical tests by observing 150 bp amplicons.

### Antimicrobial susceptibility

Antimicrobial resistance has emerged as a significant concern for public health on a global scale. The World Health Organization has identified antimicrobial resistance as one of the foremost challenges to global health in 2019^17^. Considering the importance of antibiotic resistance of *P. aeruginosa*, the antibiotic resistance patterns, and rates were evaluated in the burn patients tested and are shown in Supplementary Fig. S1. Fifty-six percent of the isolated strains were XDR and 41% were MDR. The highest anti-microbial resistance rate was related to piperacillin (80%) and levofloxacin (79%), and the least rate was for amikacin (23%).

### Phage isolation

After phage enrichment of 10 samples collected from municipal and hospital effluents, 4 phages were isolated. The isolated phages obtained from both sources (municipal and hospital effluents) produced clear plaques with different sizes and phage enzymatic activities. Based on the phage EOP, plaque diameter, and phage enzymatic activities the phage vB_PaS-HSN4 was selected for further tests and experiments.

### Phage morphology

**Figure 1:**
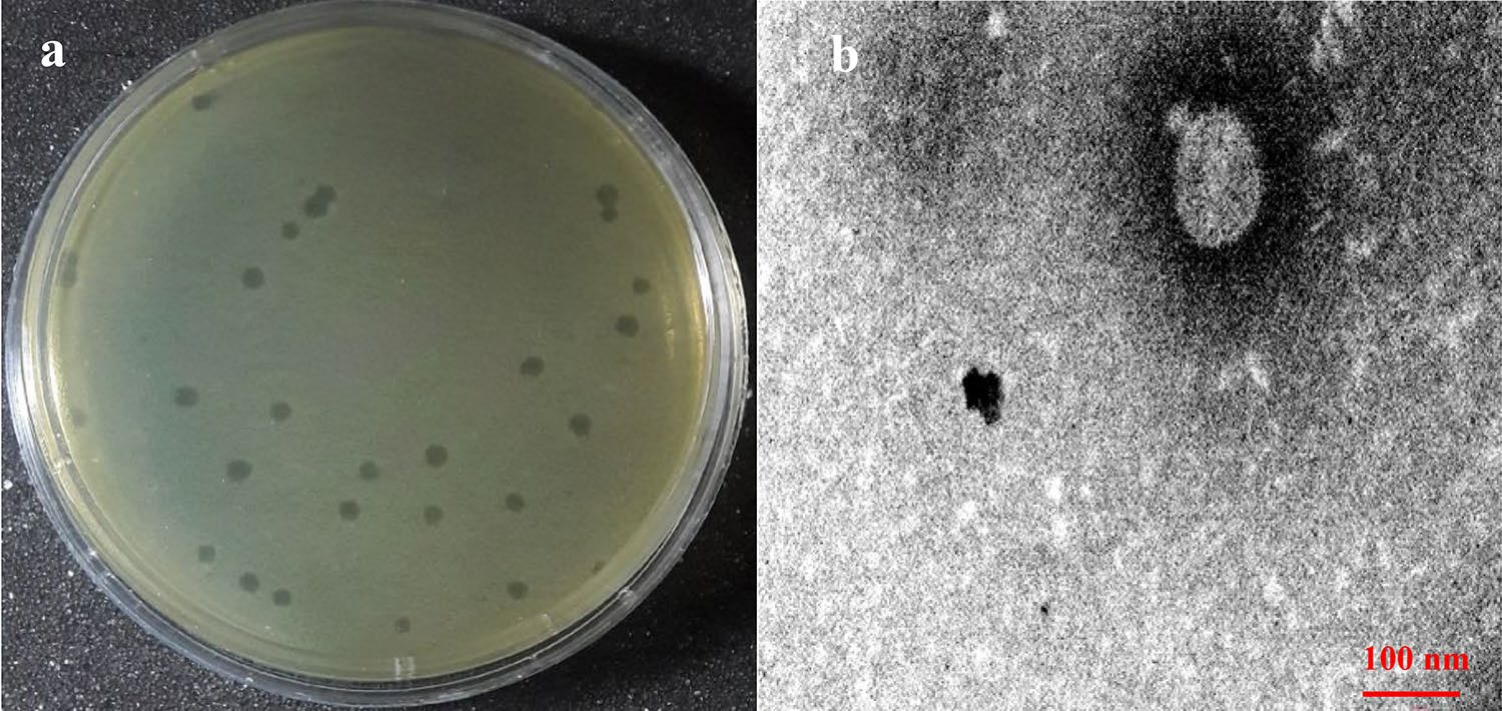
Formed plaques on *P. aeruginosa* ATCC15442 (**a**). TEM micrograph of the vB_PaS-HSN4 phage (**b**).

The phage vB_PaS-HSN4 produced clear plaques of about 1–2 mm in diameter in the double agar layer method after 24 h (Fig. 1a). In electron microscopy the phage had a small tail of ~ 19 nm in length and a head of ~ 90 × 80 nm in diameter. Therefore, phage vB_PaS-HSN4 was considered to be a member of *Caudoviricetes* class (Fig. 1b).

### Genomics of the phage vB_PaS-HSN4

**Figure 2:**
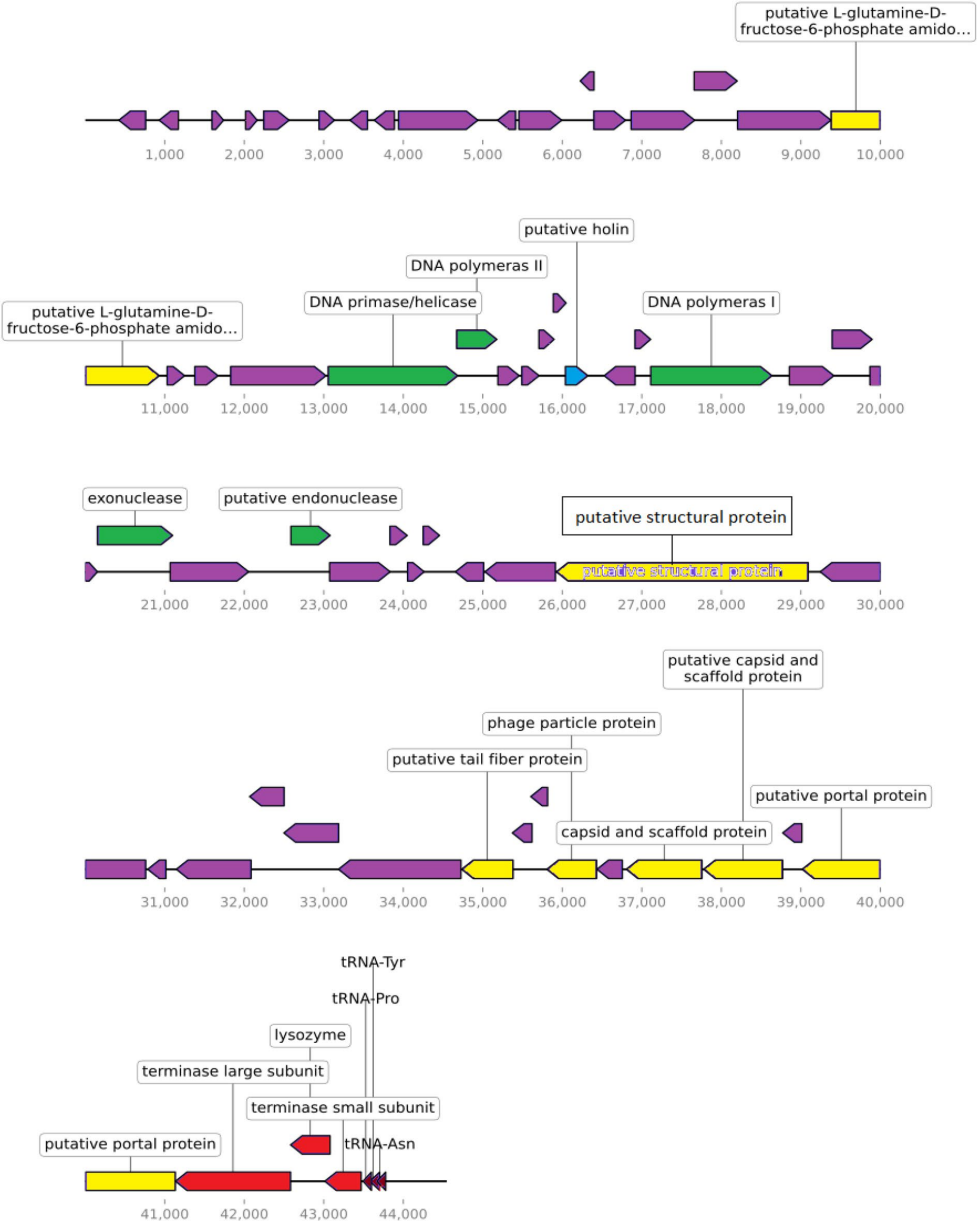
Sixty-two ORFs are shown in the genetic map of the vB_PaS-HSN4 phage using LINEAR GENOME PLOT. The function of each ORF is indicated by different colors. :DNA packaging :DNAreplication/Modification/Regulation :Structure/Morphology :Lysin/Holin.

The vB_PaS-HSN4 phage genome is dsDNA with 44,534 bp length and 53.46% G + C content. Sixty-one ORFs were identified in the phage genome (16 functional and 45 hypothetical). Twenty-seven ORFs are positioned on the complement strand and 33 on the positive strand (Supplementary Table S1). Using tRNA Scan-SE, PHIRE59, and ARNOLD servers, 3 tRNA, 5 promoters, and 7 Rho-independent transcription terminators were identified, respectively (Supplementary Table S2). According to the PhageTerm, like T5 phage, vB_PaS-HSN4 has redundant ends and DTR (PAC) phage packaging. The linear and circular genome maps are presented in Fig. 2 and Supplementary Fig. 2.

The results of comparing the complete genome of phage vB_PaS-HSN4 with 10 others using Circoletto software (Darzentas^18^), are shown in Supplementary Fig. S3. According to the diagram, the similarity of most proteins is less than 85%. The Easyfig software result shows a substantial relationship between vB_PaS-HSN4 and other *P. aeruginosa* phages within the *Bruynoghevirus* genus (Supplementary Fig. S4)^19^. Some structural and DNA replicating proteins such as amidotransferase protein (ORF17, Pf13522.9, CL0052), DNA primase/helicase (ORF21, Pf03796.18, CL0023), DNA polymerase II (ORF22, Pf01612.23, CL0219), DNA polymerase I (ORF30, Pf00476.23), exonuclease (ORF34, Pf01367.23, CL0464) and endonuclease (ORF 36, Pf05367.14, CL0236) are positioned on the positive strand. Other ORFs including tail fiber protein (ORF50, Pf07484.15, CL26890), capsid and scaffold protein (ORF 55, Pf17236.5, CL0373), terminase large subunit (ORF 59, Pf03237.18, CL0023) and lysozyme (ORF 60, Pf00959.22, CL0037) are located on the complementary strand. All protein specifications are mentioned in the Supplementary Table S1. The ARDB databases^20^ and ResFinder 4.0^21^ results showed that vB-PaS-HSN4 has no virulence-associated genes in its genome. According to the phylogenetic tree results, based on the DNA polymerase and portal protein, the vB-PaS-HSN4 phage produced a new branch in the same clade with phages Delta and Pa223 in *Caudoviricetes* class. Another phylogenetic tree, based on a terminase large subunit, also indicated that vB-PaS-HSN4 phage has the closest relationship with Delta phage (Supplementary Fig. S5a–c). Therefore, the vB_PaS-HSN4 was considered an unclassified species of the *Bruynoghevirus* genus of *Caudoviricetes* class at NCBI (Accession No. LC648443.1).

### The host range of vB_PaS-HSN4

According to the host range test, the isolated phage had lytic activity on the standard strain of *P. aeruginosa* ATCC15442 and 53.3% of the 90 clinical isolated strains including XDR and MDR *P. aeruginosa*. This phage was specific for *P. aeruginosa* and did not have effects on the other standard strains tested (Supplementary Table S3).

### pH and temperature stability of the vB_PaS-HSN4 phage

By examining phage stability at various pH values for 2, 4, 6, 8, 10, 12 and 24 h of incubations at 37 °C, the highest activity was observed at pH 8 after 2, 4, 6, 8, 10, 12 and 24 incubation hours with no significant difference (*P* ≤ 0.05). The phage was mostly stable at alkaline pH values while at pH values 2 to 6, it had less stability (Supplementary Fig. S6). Phage vB_PaS-HSN4 had its highest activity at 25 and 37 °C after 2, 4, 6, 8, 10, 12 and 24 h of incubation, with no significant difference (*P* ≤ 0.05). While the temperature escalated, the bacteriophage activity began to decrease so that it was completely inactivated at 70 °C after one incubation hour (Supplementary Fig. S7).

### The phage salt stability

The phage activity was decreased by increasing the time in all the concentrations tested. The highest phage activity was detected for 1% and 5% saline concentrations. After 6 h, compared to the first hour, in salt concentrations of 10% and above, the decrease in phage titer was significant (*P* ≤ 0.05) (Supplementary Fig. S8).

### Phage adsorption rate and cationic ions

Ca^2+^ ions may alter the ligand binding site of the receptors of the bacteriophages on the cell surface and increase the attachment of more phages to the bacterium and also transfer of the phage nucleic acids^22^. The adsorption rate of vB_PaS-HSN4 to its bacterial host in the control group was equal to 81.5% after 5 min. While in the phage mixture, containing MgCl_2_ or CaCl_2_ it was 92.7% and 95.4%, respectively. The highest phage absorption rate was observed after 15 min, 98.9% in the control group and 99.94% and 99.95% in the phage groups containing magnesium chloride or calcium chloride, respectively (Supplementary Fig. S9). According to the positive effect of MgCl_2_ or CaCl_2_ in the attachment of phage to their receptors on bacterial surfaces, due to electrostatic interactions, it seems in initial times (5 min) the attachment of vB_PaS-HSN4 to its receptors is accelerated.

### EOP (Efficiency of plating), one-step growth, and MOI of the phage vB_PaS-HSN4

**Figure 3:**
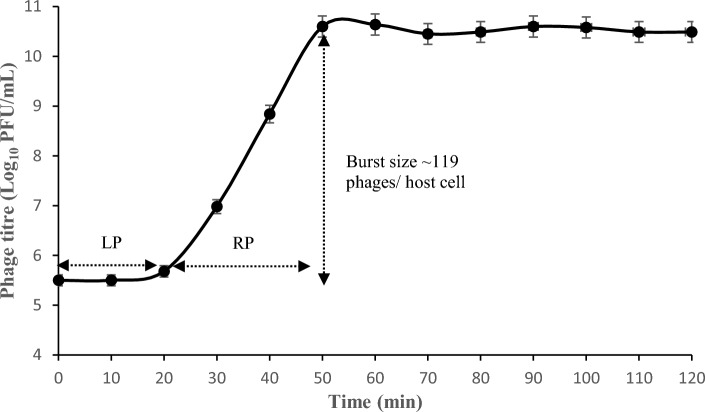
One-step growth test. LP (latent period), RP (rise period), and burst size. The results show the mean of 3 experiments. Bars: standard deviation.

**Figure 4:**
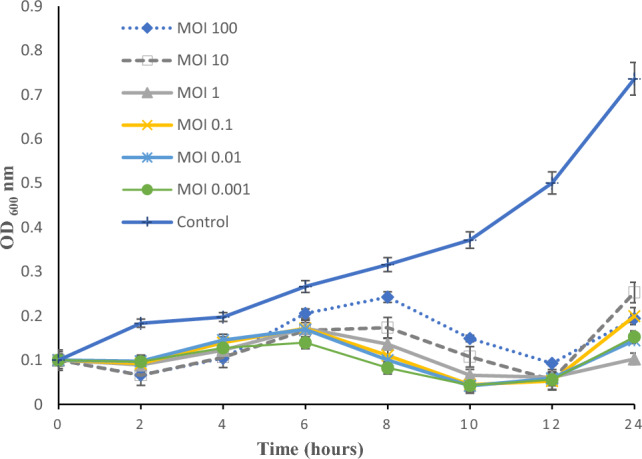
MOIs results of the vB_PaS-HSN4 phage for *P. aeruginosa.* The results show the mean of 3 experiments. Bars: standard deviation.

The vB-PaS-HSN4 phage was specific for its host *P. aeruginosa*. According to the EOP test, it was effective against 53.3% of *P. aeruginosa* strains, including XDR and MDR strains as well as *P. aeruginosa* (ATCC 15442). A high EOP was observed for 68.75% (33 of the host isolates) (> 0.5). Also, 22.91% (11) and 8.33% (4) of the other isolates had EOP of 0.2–0.5 and 0.2–0.001, respectively. The phage one-step growth curve is shown in Fig. 3. The phage has a comparatively short latent period that lasts about 20 min, and its progeny production rate (burst size) is about 119 phage particles for each infected cell. The amount of absorption after 24 h in the wells treated with MOIs of 1, 0.01, and 0. 001 was lower than the amount observed in the wells dosed with MOIs of 0.1,10, and 100, while in the early hours, it was almost the opposite. A decline in the growth of *P. aeruginosa* was observed at 8 and 10 h with MOIs 0.1, 0.01, and 0.001. The uppermost activity was detected at MOI 1 after 24 h (Fig. 4).

### Susceptibility of planktonic cells of *P. aeruginosa* to vB_PaS-HSN4

**Figure 5:**
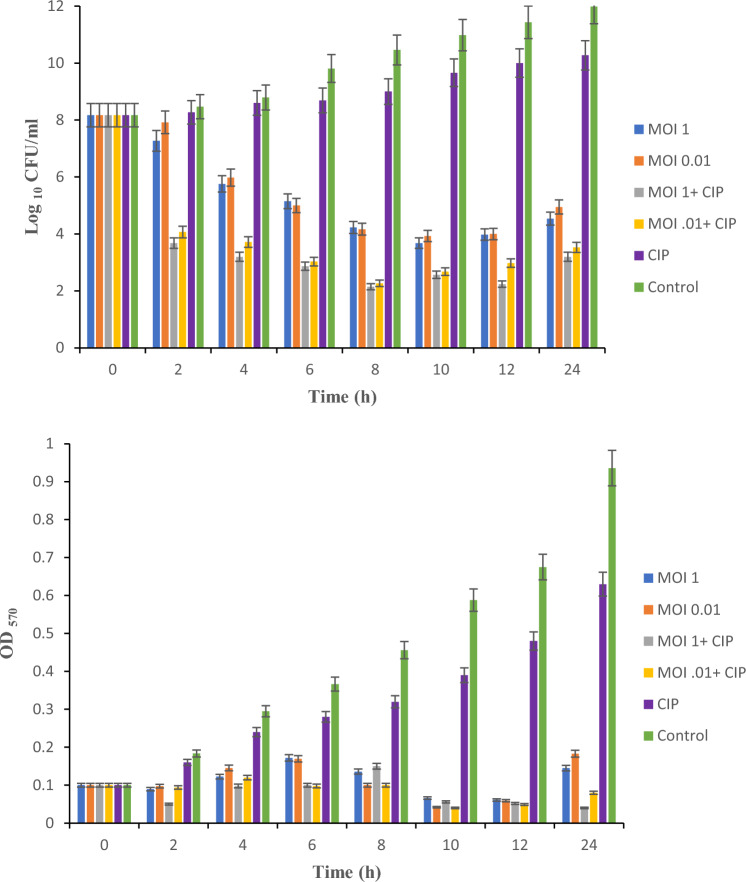
Plankton cells of *P. aeruginosa* susceptibility to vB_PaS-HSN4 and in combination with ciprofloxacin. Changes in the amount of planktonic cell CFU/ml and OD according to time in the treatment with phage, ciprofloxacin (CIP), and the mixture of the phage and CIP. Two-way ANOVA (*P* ≤ 0.05). Tests were carried out 3 times. Bars: standard deviation.

The results showed that the phage at MOIs of 1 and 0.01 reduced the bacterial cell count by approximately 4 log_10_ after 8 h, which was significant compared to the control (*P* < 0.05). After 10 h, an increase in the bacterial quantity was observed (at MOIs of 1 and 0.01). The phage in combination with sub-MIC concentration (0.5 µg/ml) of ciprofloxacin revealed that the use of phage along with the antibiotics accelerates the process of preventing the growth of the bacterium. The use of the phage and antibiotics after 2 h significantly (*P* < 0.05) reduced the bacterial count by about 4 log_10_ (Fig. 5). Also, the optical absorption of the bacterium when treated with the phage, antibiotic, and the phage combination with antibiotic was read by ELISA reader (OD_570_ nm), and the same pattern of changes were observed by OD measurements (Fig. 5).

### Biofilm removal

**Figure 6:**
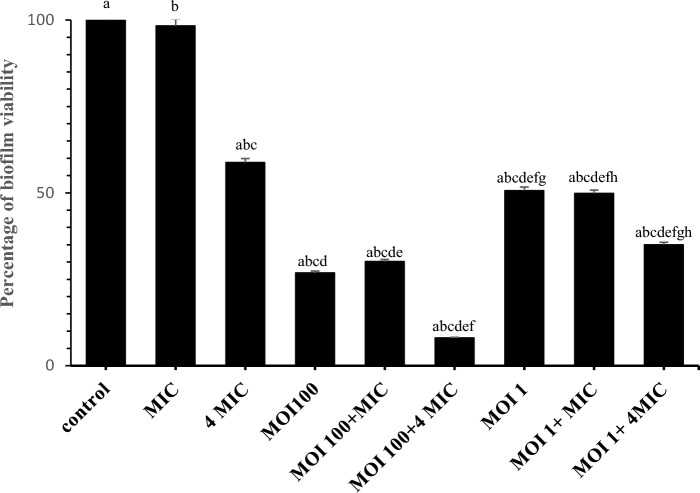
Biofilm removal test. Similar letters on each of the columns indicate a significant difference. Two-way ANOVA (*P* ≤ 0.05). Tests were carried out 3 times. Bars: standard deviation.

Compared to the control, after 24 h, vB_PaS-HSN4 phage with MOIs 1 and 100 significantly removed biofilm by 49% and 73% respectively (*P* < 0.05). Also, compared to the control, ciprofloxacin at a concentration of 4MIC (4 µg/ml) alone significantly lessened the biofilm by 42% after 24 h (*P* < 0.05). The low concentration of the antibiotic had almost no effect on biofilm removal and removed about 2% of the biofilm. The combination of the phage (MOI100) with antibiotic (4MIC) was able to significantly remove the biofilm by 92% after 24 h (*P* < 0.05). This was 65% for phage with MOI1 and antibiotic with 4MIC concentration (Fig. 6).

### Phage therapy trail in a *Galleria mellonella* infection mock-up

**Figure 7:**
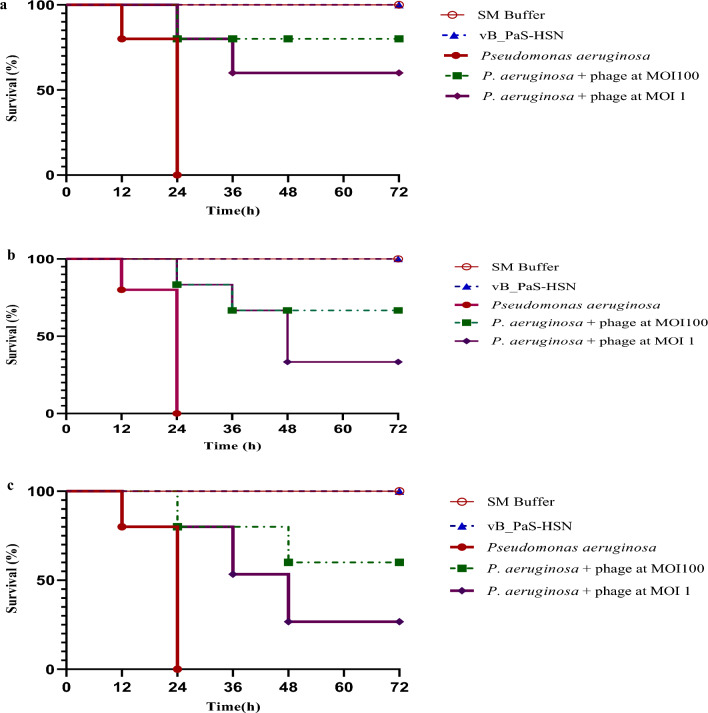
The survival rate of *G. mellonella* larvae that were infected with *P. aeruginosa* and treated with vB_PaS-HSN4 phage in the pre-treatment (n = 25) (**a**), co-treatment (n = 25) (**b**), and post-treatment (n = 25) (**c**) experiments (including five different treatment groups each containing five larvae). In all the investigated groups, a significant difference between the survival levels of the phage-treated groups (at MOIs of 1 and 100) and the groups infected with the bacterium was observed. Tests were carried out 3 times and data were analyzed by log-rank (Mantel-Cox) test.

*Galleria mellonella* larvae were employed as an animal mock-up to investigate the therapeutic effect of phage vB_PaS-HSN4 against a strain of *P. aeruginosa* that was isolated from burn infection. The log-rank (Mantel-Cox) test was employed for survival curves. No mortality was detected in the control groups after 72 h and 100% of the larvae survived. In the groups receiving the bacterium alone, after 24 h, 100% mortality was observed. In the pre-treatment group (Fig. 7a), which received the phage one hour before the injection of the bacterium, after 72 h, the percentage of surviving larvae at MOI 100 and MOI 1 was significantly improved to 80% and 60% (*P* < 0.0001), respectively. In the group that was injected with the phage and the bacterium simultaneously (co-treatment group) with the same MOIs (Fig. 7b), the percentage of surviving larvae was significantly improved to 67% and 33% (*P* < 0.0001) after 72 h. Also, in the post-treatment group results for MOI 100 and MOI 1 were significantly improved to 60% and 26% (*P* < 0.0001), respectively (Fig. 7c). The highest phage activity was observed in the pre-treatment experiment.

## Discussion

*P. aeruginosa* especially XDR strains are a serious threat in the hospital environment and are frequently isolated from wound infections, burns, and pneumonia^23^. Carbapenem-resistant *P. aeruginosa* has been designated by WHO as a critical or “priority 1” pathogen in the considerable necessity of new treatments to counter this impending public health dilemma of resistance^24^. The vB_PaS-HSN4 phage is a new lytic phage (an unclassified species of *Bruynoghevirus* genus of *Caudoviricetes* class of the *Urovicota* phylum). This phage is specific for *P. aeruginosa* and had no effect on other Gram-negative and positive strains tested, which is one of the positive features of this phage as a suitable alternative for the cure of MDR and XDR *P. aeruginosa* strains. Another important property of a phage to be used for phage therapy is having a short latent period and large burst size. vB_PaS-HSN4 had a longer latent period (20 min) and higher burst size (119 phages/host cell) compare to vB-PaeP-007 phage (latent period = 10 min, burst size = 93 phages/host cell)^25^, but had a shorter burst size and latent period compare to JGO24 (burst size = 180 phages/host cell, latent period = 50 min)^26^. Therefore, different isolated phages vary regarding their latent periods and burst sizes and among them, vB_PaS-HSN4 is a suitable nominee for phage therapy.

As it is shown in Fig. 3 the titer of the bacterium was decreased for up to 10 h with different MOIs that seems there is no resistance, but then after the number of the bacteria increases. The selection of some of the bacteria that phage did not absorb to them and overpopulation of them and also the emergence of mutated strains could be the reason for this phenomenon^27,28^. This is a critical issue and vital to tackle it. Using a phage cocktail is an effective strategy to effectively inhibit and control the possible emergence of phage resistant phenotypes^29–31^. Also, simultaneous use of bacteriophages and antibiotics is another approach to solve this issue.

In this study, the effect of the phage in combination with the antibiotic ciprofloxacin on planktonic cells and biofilm removal was investigated. Examining the sensitivity of planktonic cells to the phage alone (MOI 1 and 0.01), the combination of the phage and antibiotic in sub-MIC concentrations revealed that the antibiotic and phage combination significantly (*P* < 0.05) lessened the bacterial cells number by 4 log_10_ after 2 h, while the phage alone significantly (*P* < 0.05) lessened the bacterial cells number about 4 log_10_ after 8 h. The mechanism of ciprofloxacin's impact on bacterial DNA synthesis is influenced by the disruption of DNA gyrase subunit A function. This disruption leads to the inhibition of DNA synthesis and replication within the host cell. Nevertheless, bacteria possess certain mechanisms to counter the effects of ciprofloxacin, primarily through mutations in the DNA gyrase^32^. The effects of ciprofloxacin on phage DNA replication are intricate. Phages that harbor DNA gyrase can be suppressed by ciprofloxacin's action^33^. Conversely, phages lacking DNA gyrase subunits remain unaffected by ciprofloxacin. Even when exposed to concentrations of 1 × or 3 × MIC^34^. According to the phage genome sequencing, vB_PaS-HSN4 phage lacks the genes related to DNA gyrase which explains the synergism observed in this study.

The highest amount of biofilm removal was observed in the combination of ciprofloxacin with a concentration of 4 MIC and the phage (MOI100) so that after 24 h. The biofilm was removed by 92%, while this value for the combination of the phage with the same MOI and antibiotic with one MIC concentration was 70%. An investigation by Engeman et al.^35^, also showed that the relationship between phage and the antibiotics ceftazidime, meropenem, and ciprofloxacin is synergistic. Moreover, in the investigations conducted by Holger et al.^36^ and Chang et al.^37^, the synergism of EM and PEV20 phages in combination with ciprofloxacin has been confirmed.

Along with the role of DNA gyrase, it has already been shown that the action of ciprofloxacin is restricted to the air-biofilm interface areas, and bacterial filamentation is observed, while the bacteria in the interior sections are not affected. The lack of oxygen in the mid-layer of the biofilm is the most probable cause of the decrease in bacterial metabolic activity^37^. Phages also destroy the biofilm matrix backbone including extracellular polymeric substances (exopolysaccharides)^12^ and reduce extracellular integrity and expose metabolically inactive bacteria to the nutrients in the media and activate them. This facilitates the access of both phage and ciprofloxacin to the bacterium and hence their antimicrobial effects. Moreover, phages can remain alive, diffuse, and multiply in the biofilm matrix. The proximity of the phage and bacterial cells inside the biofilm is an advantage for phage to multiply and produce high local titers^37^. Therefore, the combination therapy of the phage with ciprofloxacin is suggested.

The vB_PaS-HSN4 phage was found to be tolerant to temperatures varying between 4 and 60 °C but was inactivated at 70 °C. These are compatible with those reported for AZ1 and MA-1 phages of *P. aeruginosa* ^16,38^.

The stability and infectivity of phage at different pH values is an important characteristic. In healthy skin, pH values range from 4.2 to 5.6, and in second-degree burns the mean initial pH of the burns was 8.55 (ranged 6.5–9)^39^. The approximate pH of wounds infected with *P. aeruginosa* is > 8.5^40^. An alkaline environment increases the growth and virulence of *P. aeruginosa*^41^*.* Moreover, the pH also affects the effectiveness of the antibiotics. The effectiveness of the aminoglycosides and macrolides is decreased in alkaline environments. On the other hand, the activity of beta-lactams increases in acidic conditions^42^. vB_PaS-HSN4 phage is active in a broad span of pH values from 4 to 12 and is more active in alkaline conditions (7–12). Therefore, it has no limitation of activity reported for antibiotics. This is also an advantage for this phage to be considered for phage therapy for infected burns.

CaCl2 and MgCl2 can stabilize the interplay between the bacteria and the phage (receptor and ligand) due to electrostatic interactions^16^. This affects absorption and penetration and other stages of the phage propagation. The infectivity of the vB_PaS-HSN4 phage was slightly increased with 10 mM CaCl_2_ and MgCl_2_ concentration. These results are compatible with earlier studies highlighting that Ca^++^ and Mg^++^ increased the adsorption of the phage compared to the control^16,38^.

The results of vB_PaS-HSN4 genome analyses indicated that it belongs to the *Caudoviricetes* class, like pa223 (Identity: 93.16%, Query coverage: 87%, Accession No. MK837012.1, Size: ~ 45 kbp, host: *P. aeruginosa*)^43^ and TL (Accession No. HG518155.1, Size: ~ 45 kbp, host: *P. aeruginosa*)^44^ phages. The phylogenetic tree analyses based on portal protein, DNA polymerase, and terminase large subunit indicated that vB_PaS-HSN4 phage has the closest relationship with phages pa223 (Accession No.MK837012.1)^43^ and Delta (Accession No. MG432151.1)^45^ that were isolated against *P. aeruginosa*. Comparing the phage main proteins revealed that DNA primase/helicase (ORF21) was 97% similar to *P. aeruginosa* phage PSA31^46^. ORF30 which is related to the vB_PaS-HSN4 phage DNA polymerase I had 98.34% similarity to *P. aeruginosa* phage Delta^45^. Capsid coding protein (ORF56) was 98% similar to *P. aeruginosa* phage pa223^43^. Moreover, portal protein (ORF58) had 97% similarity to *P. aeruginosa* phage pa223^43^. Terminase large subunit (ORF59) and terminase small subunit (ORF61) had 99.13% and 99% similarity to *P.aeruginosa* phage PaP3^47^ and Delta^46^, respectively. Therefore, overall, the vB_PaS-HSN4 phage can be regarded as an unclassified species of the *Bruynoghevirus* genus of *Caudoviricetes* class at NCBI (Accession No. LC648443.1).

No virulence factor was observed in the vB_PaS-HSN4 phage genome, which is one of the superiorities of a phage for use in phage therapy.

## Conclusion

Overall properties of the vB_PaS-HSN4 phage indicate it is a proper nominee to be used for phage therapy. The wide range of variation in host resistance and specific related phages, and different environmental and wound conditions limited the comprehensive study of the phage.

Since the wound infection is multifactorial and due to the narrow host specificity of the phages control of infection in the infected burn wounds needs a cocktail of phages and in some instances combination with proper antibiotics. On the other hand, the presence of inflammatory factors and also the possible presence of immune response against the phages used and vice-versa that can affect the efficiency of the phage therapy should be investigated which were beyond the scope of this study. For mimicking the burn wound, the model on *Galleria mellonella* larvae that was recently explained^48^ is suggested. Considering that *P. aeruginosa* has the ability to invade epithelial cells^49^ that can protect the bacterium from being attacked by the phage, the combination with a proper antibiotic for clearance of the bacterium in *Galleria mellonella* infection model and also in the other phage therapy experiments is suggested. Moreover, to achieve more reliable results the use of a proper laboratory animal burn model and investigation of the molecular mechanisms of the synergism of ciprofloxacin and the phage should be beard in mind.

## Materials and methods

### Bacterial isolation

This study was carried out in accordance with the Helsinki Declaration (Ethical Principles for Medical Research Involving Human Subjects), and approved by the University of Isfahan ethics committee (IR.UI.REC.1402.032). Informed consents were obtained from all patients.

A total of 100 swab specimens of wound were collected from different parts of the bodies (by swiping an area of 1 cm^2^ where the burn severity was the highest) in completely sterile conditions of patients with different age groups and genders suffering from types 2 and 3 burns who were admitted to the Emam Musa Kazem hospital in Isfahan from May 2018 to September 2018, and cultured in blood, Muller-Hinton and MacConkey agars using the spread plate method. They were, then, incubated at 37 °C under aerobic conditions overnight. Furthermore, Gram staining was conducted to differentiate between gram-positive and gram-negative bacteria, and microscopic examination was performed to assess the morphology, color, and shape of the microorganisms. The first recognition of the collected strains was depending on biochemical tests (Gram staining, catalase, and oxidase tests, reaction in TSI medium (Merck Co, Germany) and SIM medium (Merck Co, Germany), growth in Simon citrate medium (Merck Co, Germany), cetrimide agar (Quelab Co, Canada), growth at 42 °C and Oxidation/Fermentation (OF) test). Molecular confirmation of the isolates was done using specific primers for the oprL gene (R-1(5′TGCGATCACCACCTTCTACTTC-3′) and R-2 (5′-CGCTGACCGCTGCCTTTC-3′))^50^. Brain Heart Infusion (BHI) medium (BioLife Co, Italy) was used for the cultivation of all strains and stored at − 70 °C till subsequent use. The PCR program used is shown in Supplementary Table S4.

### Antibiotic sensitivity assay

The choice of antibiotics and the determination of antibiotic sensitivity were investigated based on the instructions of the Clinical Laboratory Standards Institute (CLSI) and using the disc diffusion method in Muller Hinton agar medium (Quelab Co, Canada)^51^. For this purpose, discs of different classes of antibiotics were used based on the CLSI table. Antibiotics used included: tobramycin (10 µg), ceftazidime (30 µg), cefepime (30 µg), gentamicin (10 µg), piperacillin (100 µg), meropenem (10 µg), amikacin (30 µg), aztreonam (30 µg), ciprofloxacin (5 µg), piperacillin-tazobactam (100/10 µg), imipenem (10 µg), and levofloxacin (5 µg). Non-susceptibility to at least one antibiotic in three or more antimicrobial categories was defined as MDR. Non-susceptibility to at least one antibiotic in all except for 1 or 2 antimicrobial categories was considered as XDR. Isolate number 6 with intermediate resistance to ciprofloxacin and resistant to other antibiotics was selected for further studies^52^.

### Bacteriophage isolation and propagation

Sewage samples including municipal and hospital effluents were used to isolate the phage. For this, one ml of bacterial overnight cultivate was overloaded to BHI broth culture medium (20 ml) at twice the concentration and subjected to 2 to 3 h of incubation at 37 °C. Then 20 ml of sewage samples were added to the bacterial suspensions. After 24 h of incubation, the suspensions were centrifuged at 10,000 g for 10 min and the supernatants were filtrated using a 0.45 μm microporous filter (Orange, Germany). Then, 10 μl of the filtered suspensions and 90 μl of the bacterial strains were mixed with 0.7% liquefied top soft agar and poured down on a plate of BHI agar. Plaques were recognized after 24 h of incubation at 37 °C. One plaque was collected and mixed with the suspension of the bacterium (20 ml) and then subjected to 24 h of incubation at 37 °C. To obtain pure stock phage, the two-layer agar technique was repeated 3 times^53^.

### pH and thermal stability

To assess the thermal stability of the isolated phage, 200 μl of the suspension of the phage (1 × 10^10^PFU/ml) was incubated in different time intervals (2, 4, 6, 8, 10, 12 and 24 h) at different thermal conditions (4, 25, 37, 42, 50, 60, and 70 °C) and then the phage titer was determined by two-layer agar method. To do the pH stability test, 100 μl of the phage suspension with a specific titer (1 × 10^10^PFU/ml) was surcharged to 900 µl of SM buffer at various pH values (2, 3, 4, 5, 6, 7, 8, 9, 10, 11, 12) and incubated at 37 °C. At 2, 4, 6, 8, 10, 12 and 24 h intervals 100 μl of the suspensions were removed and the phage titers were specified immediately employing the two-layer agar method^54^.

### The effects of various concentrations of NaCl on the phage stability

For this, 100 μl of the phage (1 × 10^10^PFU/ml) was surcharged to 900 μl of every different salt concentration (1%, 5%, 10%, 20%, 30%, and 40%). Then, serial dilution of every saline concentration was made after 2, 4, 6, 8, 10, 12 and 24 h, and the phage titer was specified by the two-layer agar method^55^.

### The effects of Ca +  + and Mg +  + on phage adsorption

In order to evaluate the adsorption rate in the presence of calcium and magnesium ions, BHI broth culture medium (25 ml) containing bacterial suspension in logarithmic phase with or without (control) a final concentration of 10 mM of either CaCl2 or MgCl2 was inoculated with 100 μl of the phage suspension with a titer of 1 × 10^10^PFU/ml and subjected to incubation at 37 °C. Then at specified intervals (0, 5, 10, 15, and 20 min), 500 μl of the suspension was picked up and subjected to centrifugation at 10,000 g for 5 min. Subsequently, unabsorbed phage particles were determined in the media by the two-layer agar method. The formula ʺN/N0 × 100˝ was employed to obtain the percentage of free or unabsorbed phages. N was equal to the free phages number in PFU/ml at the mentioned times and N0 was equal to the free phages number at the first time or zero time^16^.

### The multiplicity of infection (MOI)

One hundred and ninety microliters of the bacterial suspension prepared in BHI broth culture medium with turbidity equal to half McFarland was poured into a 96-well microtiter plate. Then 10 μl of different phage titers were added to each well to obtain MOIs equal to 0.0001, 0.001, 0.01, 0.1, 1, and 10 and then incubated at 37 °C at various times (2, 4, 6, 8, 10, 12, and 24 h). After incubation at the mentioned times, the light absorption of the plates was read by an ELISA reader at 570 nm. This experiment was done in triplicate^53,56^.

### Host range

Phage lytic action against *P. aeruginosa* clinical strains available in the Virology Laboratory of the University of Isfahan and standard strains was investigated. In summary, first, the test strains were cultured in separate plates according to the single-layer agar method, and then 10 µl of filtered phage were placed in the plates by the spot method and subjected to 24 h of incubation at 37 °C. The production of plaques was considered positive^57^.

### EOP (Efficiency of plating)

EOP means the capability of a phage for infecting a wide range of specific host strains. For this purpose, phage-sensitive strains were cultured using a single-layer agar method. The phage was serially diluted, and the mean PFU of each strain was computed by the spot method. Finally, the EOP value was reported as follows: EOP ≥ 0.5 (high), 0.2 ≤ EOP < 0.5 (medium), and 0.001 < EOP < 0.2 (low). A standard strain of *P. aeruginosa* (ATCC15442) was utilized as control^57^.

### One-step growth

Following Yazdi et al.^53^ *P. aeruginosa* was cultured in 5 ml of BHI broth culture medium (BioLife Co, Italy) and incubated at 37 °C. Incubation continued until the middle of the logarithmic phase. The bacterial suspension was next subjected to centrifugation at 7000 g for 10 min to precipitate the bacterial cells. Next, 2 ml BHI broth was added to the bacterial pellet followed by the addition of 100 μl of the phage suspension with specific titer to obtain an MOI of 0.01. For absorption of the phage to the bacteria, incubation was performed for 15 min at 37 °C and subsequently centrifuged at 13,000 g for 1 min. The supernatant was next discarded and 20 ml of BHI broth was added to the precipitate and subjected to incubation at 37 °C. Samples were taken at 10-min intervals for 2 h and instantly titrated using the two-layer agar method.

### Bacteriophage morphology

To determine the bacteriophage morphology through electron microscopy, first, of the concentrated bacteriophage suspension 10 µl were dropped on a carbon-coated copper grade for 5 min to be absorbed (excess amounts of suspension were gently filtered off). Then, after staining the grid with uranyl acetate 2% for 1 min, it was washed using distilled water. For complete drying of the grid, it was placed at room temperature for 1 h. Finally, it was examined employing transmission electron microscopy (Zeiss EM900, Germany) at 50KV^55^.

### Phage DNA extraction

Following Soleimani et al.^58^, briefly, 16 ml pure acetone was added to 4 ml of the phage (10^10^–10^11^ pfu/ml) and shaken vigorously. Subsequently, it was centrifuged at 3000 g for 5 min. Four milliliters of lysis buffer was added to the remaining sediment and was placed at room temperature for 5 min. Then, it was centrifuged at 11000 g for 2 min. The supernatant was transferred to the spin column and centrifuged at 9000 g for one minute. Then, two steps of centrifugation with washing buffer were performed at 9000 g for one minute. Finally, the elution buffer was poured in and centrifuged at 12,000 g for 2 min.

### Sequencing and analysis of the phage genome

The complete phage genome sequencing was carried out using the Illumina Hiseq method (Novogen Co, China) and assembled by CLC Genomics Workbench^59,60^. ORFs were determined using PHAST and GeneMarks online software^61,62^. The function of the identified proteins was evaluated by the Blastp at NCBI^60^. tRNA Scan-SE and Expasy compute PI/MV tools ^63,64^ were employed to predict possible tRNAs in the phage genome as well as to determine the isoelectric pH and molecular weight, respectively. Rho-factor independent terminators and promoters were looked for by ARNOLD^65^ and PHIRE servers^66^. The phage genome map was drawn using CGView (http://wishart.biology.ualberta.ca/cgview/) and Linear Genome Plot^67,68^. To recognize virulence and antibiotic resistance genes, the ResFinder 4.0 and ARDB database^19,20^ were employed. The phage genome was compared with other *P. aeruginosa* phages registered in NCBI and the similarity was determined by Circoletto and Easyfig software^18,19^. The phylogenetic trees were drawn based on the amino acids related to the large subunit of terminase and DNA polymerase with MEGA 7 software^69^. The termini and the packaging system of the phage were specified by the PhageTerm software of the Galaxy server (https://galaxy.pasteur.fr/)^70^. According to Kropinski et al. (2009)^71^, the phage was named vB_PaS-HSN4, and its complete genome was registered in the NCBI database (Accession no LC648443.1.)

### Combination therapy of planktonic cells

To explore the impact of the phage alone and along with antibiotics on planktonic cells, first a 0.5 McFarland bacterial suspension was made at the exponential growth phase and also the concentration was measured at OD600 and by colony counting. The OD was equal to 0.08–0.1 and the calculated titer of the bacterium was 1.5 × 10^8^. One hundred and ninety microliters of the bacterium were poured into the 96-well sterile polystyrene microtiter plate wells, and then 10 µl of the phage with specific MOIs (1 and 0.01) were poured in the wells and incubated for 24 h at 37 °C. The optical absorption of the plate was read at 2, 4, 6, 8, 10, 12, and 24 h at 570 nm. To reveal the effect of the combination of the antibiotic and phage at the same time, 100 µl of the bacterium and phage mixtures with specific MOIs (1 and 0.01) were poured into the 96-well sterile polystyrene microtiter plate wells. Then 100 µl of ciprofloxacin with sub-MIC concentration (0.5 µg/ml) was surcharged to the wells and next subjected to 24 h of incubation at 37 °C. The optical absorption of the plate was read at 2, 4, 6, 8, 10, 12, and 24 h at 570 nm. To count the number of living bacteria, 10 µl were removed at the mentioned times and dilution series was prepared. Then, 10 µl of each dilution were cultured and incubated for 24 h. Finally, the quantity of live bacterial cells was calculated through colony counting^53^.

### Biofilm removal

First, bacterial suspension (OD_600_ 0.08–0.1) was prepared in BHI broth. Then, 100 µl of the bacterial suspension were poured into the 96-well sterile polystyrene microtiter plate wells and subjected to 24 h of incubation at 37 °C without shaking for biofilm formation. After this, the planktonic cells were decanted and PBS was used 3 times to wash the wells. The phage (MOIs 100 and 1), ciprofloxacin (MIC (1 µg/ml), 4MIC concentrations), and combination of phage (MOIs 100 and 1) and ciprofloxacin (MIC, 4MIC concentrations) were used to treat the biofilm. At 37 °C, the plate was next subjected to 24 h of incubation. The wells were washed three times with PBS and desiccated at room temperature. Then 200μL of crystal violet (0.1%) was surcharged to the biomass and after 5 min washed again with PBS. Finally, 200μL of ethanol (70%) was poured into the wells. A Multi-Mode Microplate Reader, BioTek, USA was used to read the OD600 of the wells. The experiment was carried out in triplicate^53^.

### Phage therapy assay in a *Galleria mellonella* infection mock-up

**Figure 8:**
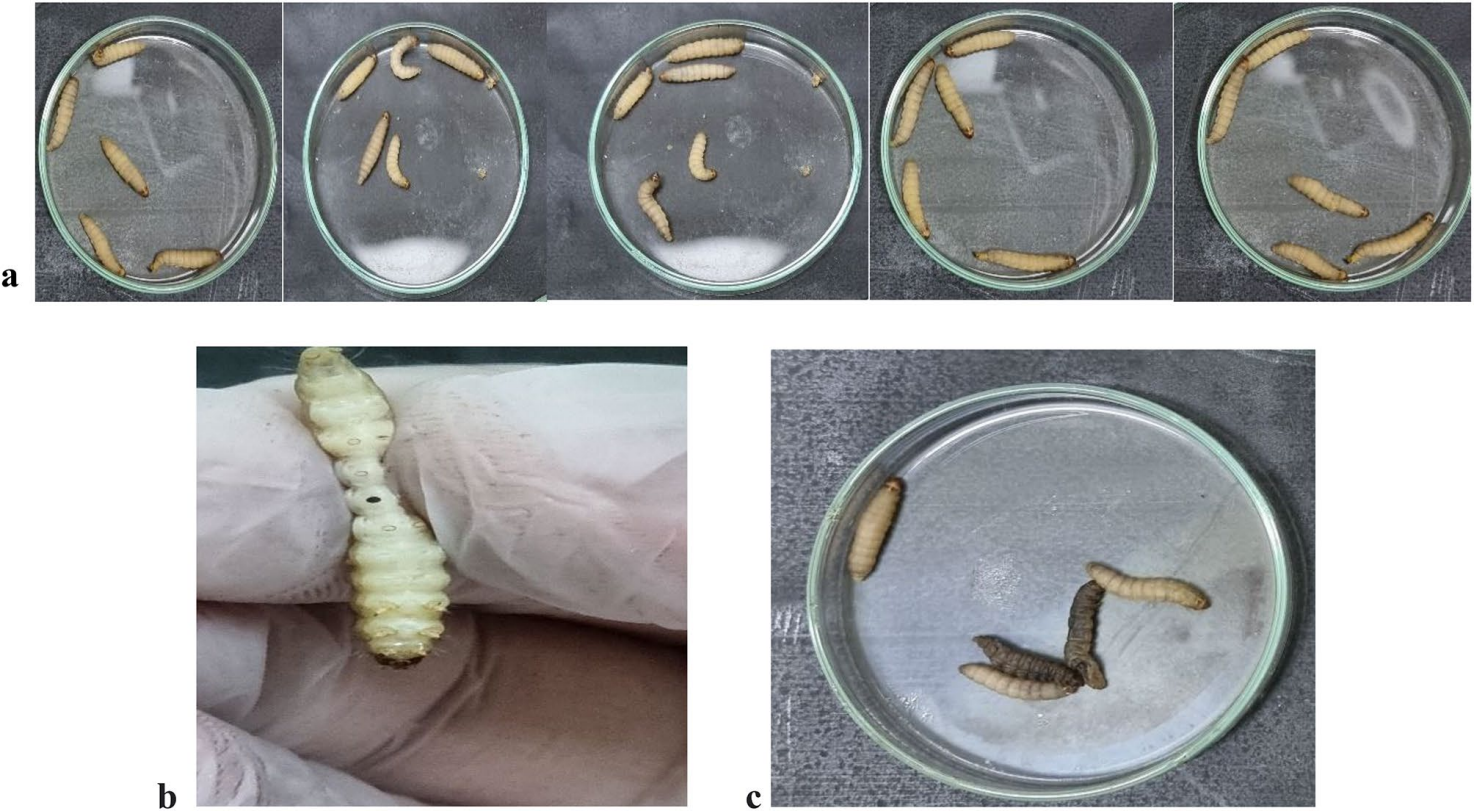
Five groups of five larvae were selected for each experiment (three replicates) (**a**), Larvae were injected at the last right-side and the left-side prolegs (**b**) and dead (dark) and survived larvae (normal) (**c**).

*Galleria mellonella* infection mock-up was employed to assess in vivo efficacy of the bacteriophage. *G. mellonella* larvae were sourced from the Isfahan Beekeeper Association and subsequently transferred to the University of Isfahan's insectarium. The larvae were maintained under optimal conditions until employed in the designed experiments. According to the method of Jeon et al. (2019), *G. mellonella* larvae weighing 180–250 mg were randomly elected. Three pre-treatment, co-treatment, and post-treatment experiments were performed each including control group 1: which received SM buffer alone, control group 2: which received vB_PaS-HSN4 phage (1 × 10^10^ PFU/ml) alone, group 3: which received the bacterium (1 × 10^8^ CFU/ml) alone, group 4: which received the bacterium (1 × 10^8^ CFU/ml) plus vB_PaS-HSN4 phage (1 × 10^8^ PFU/ml) (MOI 1), group 5: which received the bacterium (1 × 10^8^ CFU/ml) plus vB_PaS-HSN4 phage (1 × 10^10^ PFU/ml) (MOI 100). Five larvae were tested in each group (Fig. 8a). The experiments were performed in triplicate to ensure the accuracy and reliability of the results. In the pre-treatment experiment, phage was injected (in the right-side last proleg) 1 h before injecting the bacterium (in the left-side last proleg) (Fig. 8b). In the co-treatment group the phage and the bacterium were injected at the same time. In the post-treatment experiment, phage was injected (in the right-side last proleg) 1 h after the injection of the bacterium (in the left-side last proleg). The larvae were next incubated at 37 °C and their survivals were determined at 24, 48, and 72 h of incubation. The larvae were considered dead when their color turned dark and no movement in response to touch was observed (Fig. 8c)^72^.

### Statistical analyses

Data analyses were performed using T-test, One-way, Two-way, and Multiple comparisons ANOVA, and also the log-rank (Mantel-Cox) tests utilizing GraphPad Prism software (version 8.0) (GraphPad Software Inc., USA).

### Supplementary Information

Supplementary Figure S1.

Supplementary Figure S2.

Supplementary Figure S3.

Supplementary Figure S4.

Supplementary Figure S5.

Supplementary Figure S6.

Supplementary Figure S7.

Supplementary Figure S8.

Supplementary Figure S9.

Supplementary Tables.

## Data Availability

The whole genome sequence of *P. aeruginosa* phage vB_PaS-HSN4 is accessible in GenBank with accession number LC648443.1.
