# Supplementary material for: Genomic analysis of vB_PaS-HSN4 bacteriophage and its antibacterial activity (in vivo and in vitro) against Pseudomonas aeruginosa isolated from burn
Source: Sci Rep. 2024 Jan 23;14:2007. doi: 10.1038/s41598-023-50916-5 (PMC10805781; doi:10.1038/s41598-023-50916-5)
Supplement: Supplementary file 3 — Supplementary Figure S3. [file 41598_2023_50916_MOESM3_ESM.pdf]

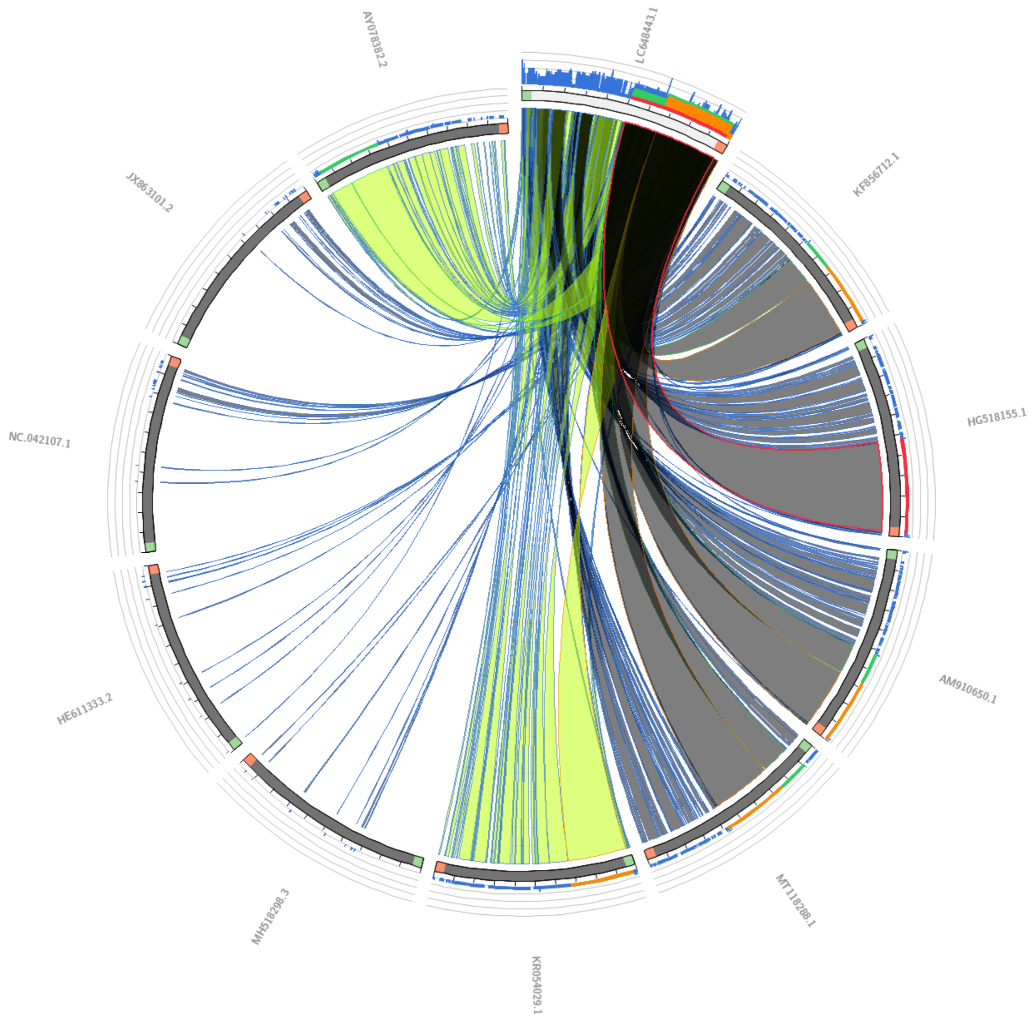

Supplementary Fig. 3. The similarity of vB_PaS-HSN4 with 10 other phages is shown. The colored lines and ribbon represent similarities of phage genomes as blue <25%, green <50, orange <75%, and red >97%.
