# Supplementary material for: Genomic analysis of vB_PaS-HSN4 bacteriophage and its antibacterial activity (in vivo and in vitro) against Pseudomonas aeruginosa isolated from burn
Source: Sci Rep. 2024 Jan 23;14:2007. doi: 10.1038/s41598-023-50916-5 (PMC10805781; doi:10.1038/s41598-023-50916-5)
Supplement: Supplementary file 4 — Supplementary Figure S4. [file 41598_2023_50916_MOESM4_ESM.pdf]

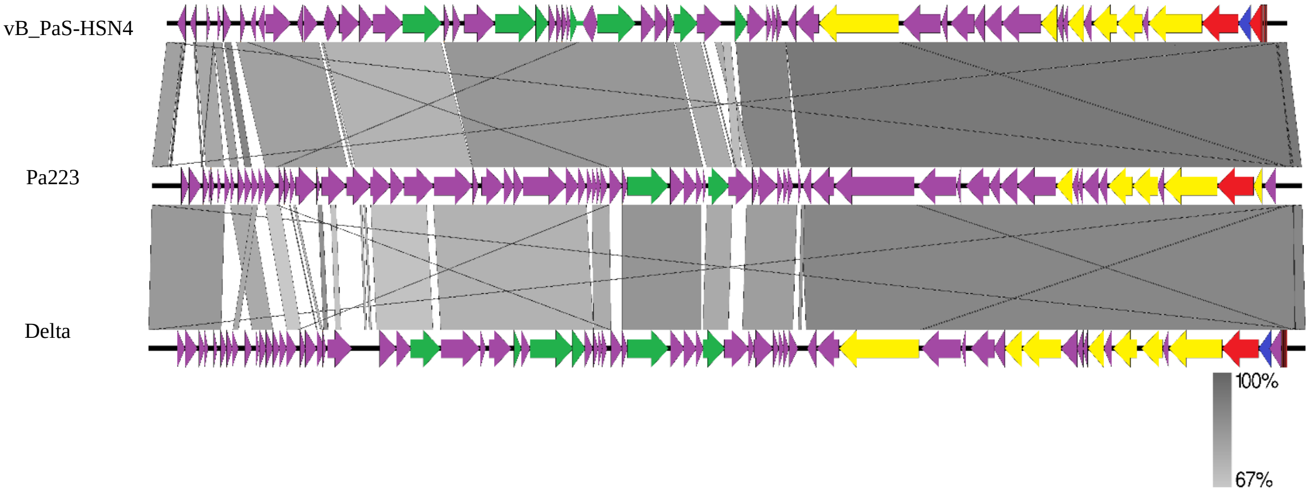

Supplementary Fig. 4. The comparison of the whole genome sequence of the vB_PaS-HSN4 phage with similar phages (pa223 and Delta) using Easy fig. The colored arrows indicate ORFs according to their predicted function. The homologous regions between phages are indicated by gray shading.

: DNA packaging : DNA replication/Modification/Regulation : Structure/Morphology : Lysin/Holin
