# Supplementary material for: Genomic analysis of vB_PaS-HSN4 bacteriophage and its antibacterial activity (in vivo and in vitro) against Pseudomonas aeruginosa isolated from burn
Source: Sci Rep. 2024 Jan 23;14:2007. doi: 10.1038/s41598-023-50916-5 (PMC10805781; doi:10.1038/s41598-023-50916-5)
Supplement: Supplementary file 5 — Supplementary Figure S5. [file 41598_2023_50916_MOESM5_ESM.pdf]

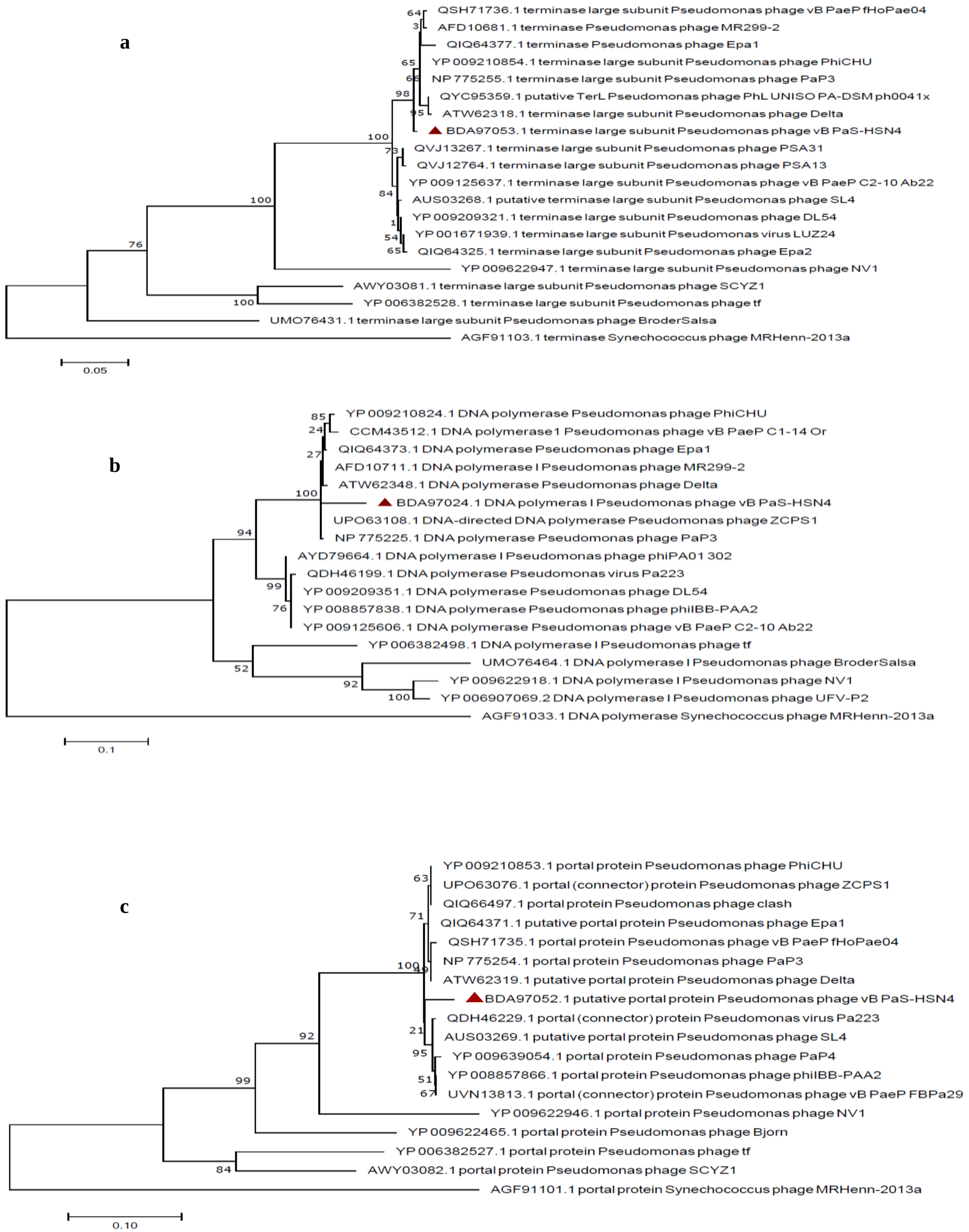

Supplementary Fig. 5. Phylogenetic trees of vB_PaS-HSN4 phage was drawn using MEGA 7 software based on the amino acid sequence of terminase large subunit (a), DNA polymerase (b) and portal protein (c). ▲: sequences of the phage detected in this study.
