# Supplementary material for: Genomic analysis of vB_PaS-HSN4 bacteriophage and its antibacterial activity (in vivo and in vitro) against Pseudomonas aeruginosa isolated from burn
Source: Sci Rep. 2024 Jan 23;14:2007. doi: 10.1038/s41598-023-50916-5 (PMC10805781; doi:10.1038/s41598-023-50916-5)
Supplement: Supplementary file 7 — Supplementary Figure S7. [file 41598_2023_50916_MOESM7_ESM.pdf]

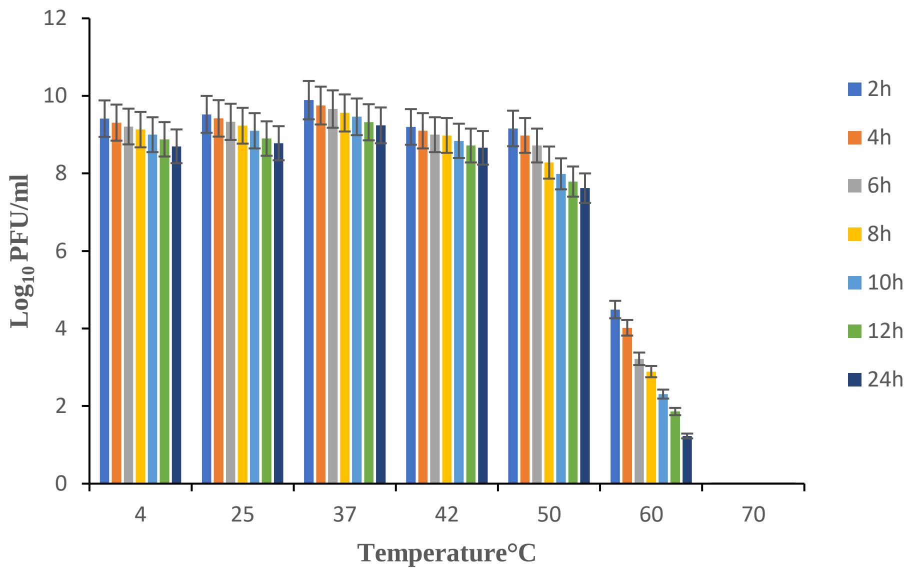

Supplementary Fig. 7. The effects of different temperatures (4 to 70 °C) on the stability of the vB_PaS-HSN4 phage. The results show the mean of three experiments. Bars: standard deviation.
