# Supplementary material for: Genomic analysis of vB_PaS-HSN4 bacteriophage and its antibacterial activity (in vivo and in vitro) against Pseudomonas aeruginosa isolated from burn
Source: Sci Rep. 2024 Jan 23;14:2007. doi: 10.1038/s41598-023-50916-5 (PMC10805781; doi:10.1038/s41598-023-50916-5)
Supplement: Supplementary file 10 — Supplementary Tables. [file 41598_2023_50916_MOESM10_ESM.doc]

**Title: Genomic analysis of VB_PaS-HSN4 bacteriophage and its antibacterial activity (*in vivo* and *in vitro*) against *Pseudomonas aeruginosa* isolated from burn**

**Solmaz Rafiei & Majid Bouzari***

Department of Cell and Molecular Biology & Microbiology, Faculty of Biological Science and Technology, University of Isfahan, Hezar-Jereeb Street, 81746-73441, Isfahan, Iran.

*Corresponding author: Professor Majid Bouzari, Department of Cell and Molecular Biology & Microbiology, Faculty of Biological Science and Technology, University of Isfahan., Hezar-Jereeb Street, 81746-73441, Isfahan, Iran. Email: [bouzari@sci.ui.ac.ir](mailto:bouzari@sci.ui.ac.ir) or [mbouzari@yahoo.com](mailto:mbouzari@yahoo.com)

**Supplementary Table 1. Phage vB_PaS-HSN4 gene annotations.**

| **ORF** | **Position (nt)** | | **Strand** | **Codon** | | **Size (aa)^1^** | **E value** | **Identity (%)** | **Closest hit (accession number)** | **Conserved protein domain family** | **Predictive Function** | **pI^3^** | **Mw^2^ (Da)** | **Accession**  **number** |
| --- | --- | --- | --- | --- | --- | --- | --- | --- | --- | --- | --- | --- | --- | --- |
|  | **From** | **To** |  | **Start** | **Stop** |  |  |  |  |  |  |  |  |  |
| 1 | 423 | 761 | - | ATG | TAG | 112 | 1e-27 | 98.04% | hypothetical protein oldone_1 [Pseudomonas phage oldone] |  | Hypothetical protein | 7.83 | 12865.81 | BDA96995.1 |
| 2 | 930 | 1172 | - | GTG | TAG | 80 | - | - | - |  | Hypothetical protein | 4.81 | 8147.26 | BDA96996.1 |
| 3 | 1595 | 1741 | + | GTG | TGA | 48 | 1e-23 | 100 | hypothetical protein [Pseudomonas phage vB_PaeP_fHoPae04] | PF11656.11 | Hypothetical protein | 11.67 | 6843.03 | BDA96997.1 |
| 4 | 2018 | 2164 | + | ATG | TGA | 48 | 4e-27 | 100 | hypothetical protein AVV32_gp13 [Pseudomonas phage PhiCHU] | PF20490.1  CL0370 | Hypothetical protein | 6.38 | 5559.39 | BDA96998.1 |
| 5 | 2246 | 2566 | + | TTG | TGA | 106 | 1e-57 | 89.90 | hypothetical protein [Pseudomonas phage PhL_UNISO_PA-DSM_ph0041x] |  | Hypothetical protein | 9.40 | 11895.58 | BDA96999.1 |
| 6 | 2941 | 3135 | + | GTG | TGA | 64 | 2e-30 | 85.94 | hypothetical protein [Pseudomonas phage SaPL] |  | Hypothetical protein | 7.08 | 6980.77 | BDA97000.1 |
| 7 | 3330 | 3351 | - | ATG | TAG | 73 | - | - |  |  | Hypothetical protein | 4.45 | 7777.59 | BDA97001.1 |
| 8 | 3635 | 3889 | - | GTG | TGA | 84 | - | - |  |  | Hypothetical protein | 4.34 | 9250.19 | BDA97002.1 |
| 9 | 3941 | 4939 | + | GTG | TGA | 332 | 2e.145 | 91.30 | hypothetical protein Pa223_017 [Pseudomonas virus Pa223] | PF01145.28  CL0433 | Hypothetical protein | 8.30 | 35325.47 | BDA97003.1 |
| 10 | 5186 | 5413 | - | ATG | TGA | 75 | - | - |  |  | Hypothetical protein | 4.67 | 7804.82 | BDA97004.1 |
| 11 | 5455 | 5994 | + | ATG | TGA | 179 | 1e-96 | 78.77 | hypothetical protein [Pseudomonas phage SaPL] |  | Hypothetical protein | 6.44 | 19943.69 | BDA97005.1 |
| 12 | 6220 | 6399 | - | ATG | TAG | 57 | - | - |  |  | Hypothetical protein | 4.51 | 5972.75 | BDA97006.1 |
| 13 | 6398 | 6796 | + | ATG | TAA | 132 | 7e-50 | 67.48 | hypothetical protein PaPSe_gp53 [Pseudomonas phage PaP_Se] | PF05891.15  CL0063 | Hypothetical protein | 5.19 | 14870.70 | BDA97007.1 |
| 14 | 6866 | 7666 | + | ATG | TGA | 266 | 1e-152 | 79.70 | hypothetical protein [Pseudomonas phage SaPL] AYJ73800.1 | PF14395.9 | Hypothetical protein | 4.81 | 29066.78 | BDA97008.1 |
| 15 | 7659 | 8201 | + | ATG | TGA | 180 | 5e-101 | 82.22 | hypothetical protein phiIBBPAA2_0021 [Pseudomonas phage  phiIBB-PAA2] YP_008857822.1 | PF01867.19 | Hypothetical protein | 5.14 | 20203.49 | BDA97009.1 |
| 16 | 8205 | 9380 | + | ATG | TAA | 391 | 0.0 | 99.74 | hypothetical protein [Pseudomonas phage SaPL] | PF12224.11  CL0286 | Hypothetical protein | 5.33 | 44543.60 | BDA97010.1 |
| 17 | 9381 | 10934 | + | ATG | TAA | 517 | 0.0 | 94.20 | putative L-glutamine-D-fructose-6-phosphate amidotransferase  [Pseudomonas phage clash] QIQ66456.1 | PF13522.9  CL0052 | putative L-glutamine-D-fructose-6-phosphate amidotransfrase | 6.19 | 56896.22 | BDA97011.1 |
| 18 | 11031 | 11252 | + | ATG | TAA | 73 | 2e-41 | 94.52 | hypothetical protein I7C_045c [Pseudomonas phage MR299-2] AFD10723.1 |  | Hypothetical protein | 4.77 | 8588.79 | BDA97012.1 |
| 19 | 11379 | 11672 | + | ATG | TGA | 97 | 1e-43 | 82.02 | hypothetical protein Epa2_gp21 [Pseudomonas phage Epa2] QIQ64333.1 |  | Hypothetical protein | 10.83 | 10767.37 | BDA97013.1 |
| 20 | 11830 | 13029 | + | ATG | TAA | 399 | 4e-72 | 84.83 | hypothetical protein Delta_p44 [Pseudomonas phage Delta] ATW62359.1 | PF06094.15  CL0278 | Hypothetical protein | 5.56 | 44063.90 | BDA97014.1 |
| 21 | 13057 | 14688 | + | TTG | TGA | 543 | 0.0 | 97.79 | DNA primase/helicase, partial [Pseudomonas phage PSA31] QVJ13229.1 | PF03796.18  CL0023 | DNA primase/helicase | 5.80 | 61960.06 | BDA97015.1 |
| 22 | 14672 | 15178 | + | ATG | TGA | 168 | 4e-121 | 99.40 | DNA polymerase 2 [Pseudomonas phage Delta] ATW62355.1 | PF01612.23  CL0219 | DNA polymerase II | 4.96 | 19695.56 | BDA97016.1 |
| 23 | 15191 | 15460 | + | GTG | TGA | 89 | 5e-53 | 96.63 | hypothetical protein I7C_039c [Pseudomonas phage MR299-2] AFD10717.1 |  | Hypothetical protein | 9.99 | 9743.54 | BDA97017.1 |
| 24 | 15492 | 15710 | + | ATG | TGA | 72 | 8e-23 | 69.12 | hypothetical protein phiIBBPAA2_0031 [Pseudomonas phage phiIBB-PAA2] YP_008857832.1 |  | Hypothetical protein | 9.40 | 8082.26 | BDA97018.1 |
| 25 | 15707 | 15898 | + | ATG | TAA | 63 | 3e-37 | 96.83 | hypothetical protein [Pseudomonas phage vB_PaeP_fHoPae04] QSH71702.1 |  | Hypothetical protein | 4.76 | 7363.24 | BDA97019.1 |
| 26 | 15888 | 16049 | + | ATG | TAA | 53 | 3e-24 | 88.46 | hypothetical protein AVV32_gp37 [Pseudomonas phage PhiCHU] YP_009210820.1 |  | Hypothetical protein | 4.04 | 5717.19 | BDA97020.1 |
| 27 | 16040 | 16324 | + | GTG | TGA | 94 | 3e-58 | 95.74 | putative holin [Pseudomonas phage SaPL] AYJ73814.1 |  | putative holin | 9.61 | 10673.54 | BDA97021.1 |
| 28 | 16527 | 16916 | - | ATG | TAA | 129 | - | - |  |  | Hypothetical protein | 9.99 | 13968.41 | BDA97022.1 |
| 29 | 16915 | 17112 | + | ATG | TAA | 65 | 1e-37 | 100 | hypothetical protein AVV32_gp40 [Pseudomonas phage PhiCHU]phage ST2] YP_009210823.1 | PF07111.15 | Hypothetical protein | 4.61 | 7494.60 | BDA97023.1 |
| 30 | 17113 | 18639 | + | GTG | TGA | 508 | 0.0 | 94.57 | DNA polymerase I [Pseudomonas phage MR299-2] AFD10711.1 | PF00476.23 | DNA polymerase I | 9.96 | 56496.45 | BDA97024.1 |
| 31 | 18857 | 19417 | + | ATG | TAA | 186 | 5e-119 | 97.85 | hypothetical protein AVV32_gp42 [Pseudomonas phage PhiCHU] YP_009210825.1 |  | Hypothetical protein | 4.86 | 20763.41 | BDA97025.1 |
| 32 | 19395 | 19898 | + | ATG | TAA | 167 | 1e-106 | 97.60 | hypothetical protein BN425_orf_30 [Pseudomonas phage  vB_PaeP_p2-10_Or1] YP_007183238.1 | PF18909.3 | Hypothetical protein | 5.73 | 18282.55 | BDA97026.1 |
| 33 | 19870 | 20154 | + | ATG | TGA | 94 | 3e-48 | 81.72 | hypothetical protein AVU27_gp30 [Pseudomonas phage DL54] YP_009209348.1 |  | Hypothetical protein | 4.50 | 10556.77 | BDA97027.1 |
| 34 | 21054 | 21101 | + | ATG | TGA | 315 | 0.0 | 98.65 | 5'-3' exonuclease [Pseudomonas phage MR299-2] AFD10707.1 | PF01367.23  CL0464 | exonuclease | 5.03 | 36407.90 | BDA97028.1 |
| 35 | 21070 | 22056 | + | ATG | TGA | 328 | 0.0 | 96.93 | hypothetical protein AVV32_gp46 [Pseudomonas phage PhiCHU] YP_009210829.1 | PF11927.11 | Hypothetical protein | 5.45 | 37553.68 | BDA97029.1 |
| 36 | 22588 | 23082 | + | ATG | TAA | 164 | 4e-73 | 90.43 | endonuclease [Pseudomonas phage PhiCHU] YP_009210830.1 | PF05367.14  CL0236 | putative endonuclease | 9.34 | 19423.26 | BDA97030.1 |
| 37 | 23075 | 23836 | + | ATG | TGA | 253 | 8e-174 | 98.42 | hypothetical protein I7C_026c [Pseudomonas phage MR299-2] AFD10704.1 |  | Hypothetical protein | 9.04 | 29281.98 | BDA97031.1 |
| 38 | 23833 | 24051 | + | GTG | TAA | 72 | 4e-43 | 95.83 | hypothetical protein Epa2_gp45 [Pseudomonas phage Epa2] QIQ64360.1 |  | Hypothetical protein | 5.56 | 8157.48 | BDA97032.1 |
| 39 | 24251 | 24264 | + | ATG | TGA | 69 | 2e-42 | 97.10 | hypothetical protein oldone_46 [Pseudomonas phage oldone] QIQ67288.1 |  | Hypothetical protein | 9.25 | 7831.13 | BDA97033.1 |
| 40 | 24251 | 24457 | + | ATG | TAA | 68 | 9e-21 | 95.59 | hypothetical protein SL4_12 [Pseudomonas phage SL4] AUS03224.1 |  | Hypothetical protein | 4.69 | 7703.68 | BDA97034.1 |
| 41 | 24656 | 25012 | _ | ATG | TAA | 118 | 3e-77 | 97.46 | hypothetical protein phiIBBPAA2_0046 [Pseudomonas phage phiIBB-PAA2] YP_008857849.1 |  | Hypothetical protein | 4.64 | 12775.38 | BDA97035.1 |
| 42 | 25027 | 25914 | - | ATG | TAA | 295 | 0.0 | 98.31 | hypothetical protein Delta_p20 [Pseudomonas phage Delta] ATW62335.1 |  | Hypothetical protein | 4.88 | 31620.39 | BDA97036.1 |
| 43 | 25926 | 29093 | _ | ATG | TAA | 1055 | 0.0 | 98.86 | putative structural protein [Pseudomonas phage oldone] QIQ67294.1 |  | putative structural protein | 5.08 | 112066.82 | BDA97037.1 |
| 44 | 29244 | 30761 | _ | ATG | TAA | 505 | 0.0 | 98.81 | hypothetical protein Pa223_054 [Pseudomonas virus Pa223] QDH46215.1 |  | Hypothetical protein | 5.67 | 53504.84 | BDA97038.1 |
| 45 | 30786 | 31016 | _ | GTG | TAA | 76 | 1e-43 | 98.68 | hypothetical protein VC51_gp55 [Pseudomonas phage vB_PaeP_C2-10_Ab22] YP_009125623.1 | PF03418  CL0095 | Hypothetical protein | 5.03 | 8767.60 | BDA97039.1 |
| 46 | 31146 | 32087 | _ | ATG | TAA | 313 | 3e-154 | 97.76 | hypothetical protein FGG60_gp56 [Pseudomonas phage PaP4] YP_009639042.1 | PF17578.5 | Hypothetical protein | 4.64 | 32097.23 | BDA97040.1 |
| 47 | 32068 | 32502 | _ | ATG | TAA | 144 | 7e-102 | 97.92 | hypothetical protein FGG60_gp57 [Pseudomonas phage PaP4] YP_009639043.1 |  | Hypothetical protein | 4.88 | 16605.76 | BDA97041.1 |
| 48 | 32499 | 33188 | - | ATG | TGA | 229 | 2e-133 | 92.58 | hypothetical protein [Pseudomonas phage PhL_UNISO_PA-DSM_ph0041x] QYC95370.1 |  | Hypothetical protein | 4.85 | 25148.73 | BDA97042.1 |
| 49 | 33185 | 34726 | _ | ATG | TGA | 513 | 0.0 | 97.08 | hypothetical protein [Pseudomonas phage SaPL] AYJ73835.1 | PF13344.9  CL0137 | Hypothetical protein | 5.71 | 57567.79 | BDA97043.1 |
| 50 | 34735 | 35382 | _ | ATG | TGA | 215 | 6e-147 | 96.28 | putative tail fiber protein [Pseudomonas phage vB_PaeP_fHoPae04] QSH71727.1 | PF07484.15  CL26890 | putative tail fiber protein | 8.67 | 22179.15 | BDA97044.1 |
| 51 | 35372 | 35620 | _ | ATG | TGA | 82 | 1e-26 | 96,34 | hypothetical protein Pa223_061 [Pseudomonas virus Pa223] QDH46222.1 | PF08650.13 | Hypothetical protein | 5.74 | 8904.22 | BDA97045.1 |
| 52 | 35604 | 35816 | _ | TTG | TGA | 70 | 6e-40 | 100 | hypothetical protein FGG60_gp62 [Pseudomonas phage PaP4] YP_009639048.1 |  | Hypothetical protein | 6.43 | 7703.01 | BDA97046.1 |
| 53 | 35806 | 36432 | _ | ATG | TAA | 208 | 1e-17 | 32 | particle protein [Pseudomonas phage SCYZ1] QBQ03021.1 |  | particle protein | 4.87 | 24086.37 | BDA97047.1 |
| 54 | 36436 | 36756 | - | ATG | TAA | 106 | 2e-45 | 67.92 | hypothetical protein FDJ28_gp06 [Pseudomonas phage Bjorn] YP_009622469.1 | PF11384.11 | Hypothetical protein | 5.65 | 12015.48 | BDA97048.1 |
| 55 | 36805 | 37758 | - | ATG | TAA | 317 | 0.0 | 99.05 | capsid and scaffold protein [Pseudomonas phage PSA31] QVJ13263.1 | PF17236.5  CL0373 | capsid and scaffold protein | 6.92 | 35011.53 | BDA97049.1 |
| 56 | 3777 | 38772 | - | ATG | TAA | 331 | 3e-152 | 99.60 | putative capsid scaffold protein [Pseudomonas phage U47] QHB49066.1 |  | putative capsid and scaffold protein | 4.34 | 37229.80 | BDA97050.1 |
| 57 | 38772 | 39014 | - | TTG | TAA | 80 | 1e-50 | 98.75 | hypothetical protein SL4_58 [Pseudomonas phage SL4] AUS03270.1 |  | Hypothetical protein | 5.11 | 9314.58 | BDA97051.1 |
| 58 | 39017 | 41134 | - | ATG | TAA | 705 | 0.0 | 97.30 | putative portal protein [Pseudomonas phage  PhL_UNISO_PA-DSM_ph0041x] QYC95360.1 |  | putative portal protein | 5.03 | 80724.17 | BDA97052.1 |
| 59 | 41135 | 42583 | - | ATG | TAA | 482 | 0.0 | 99.38 | terminase large subunit [Pseudomonas phage PhiCHU] YP_009210854.1 | PF03237.18  CL0023 | terminase large subunit | 6.08 | 54651.17 | BDA97053.1 |
| 60 | 42583 | 43080 | - | ATG | TAA | 165 | 2e-79 | 87.88 | lysozyme [Pseudomonas phage phiIBB-PAA2] YP_008857868.1 | PF00959.22  CL0037 | Lysozyme | 7.80 | 18898.35 | BDA97054.1 |
| 61 | 43013 | 43471 | - | ATG | TGA | 67 | 7e-73 | 71.81 | terminase small subunit [Pseudomonas phage tf] YP_006382530.1 |  | terminase small subunit | 5.58 | 16578.84 | BDA97055.1 |

^1^Amino acids, ^2^ Molecular weight, ^3^ pH Isoelectric.

**Supplementary Table 2. Rho independent factor of vB_PaS-HSN4**


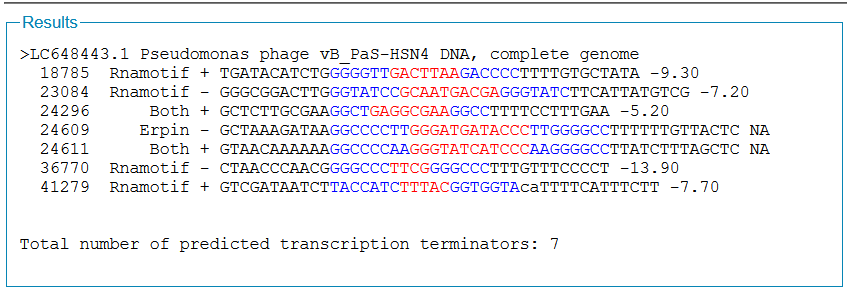


**Supplementary Table 3. Bacterial species used to assess host range.**

| Standard strains ATCC number Plaque formation |
| --- |
| *Pseudomonas aeruginosa*  ATCC 15442 +  *Enterococcus faecium* ATCC 51559 -  *Staphylococcus aureus* ATCC 25923 -  *Streptococcus agalactiae* ATCC 27956 -  *Streptococcus pyogenes* ATCC 19615 -  *Escherichia coli* ATCC 25922 -  *Staphylococcus epidermidis* PTCC 1435 -  *Staphylococcus saprophyticus* ATCC 15305 -  *Klebsiella pneumoniae* PTCC 1290 -  *Proteus mirabilis* PTCC 1710 - |

+: Presence of clear zone

-: Absence of clear zone

**Supplementary Table 4.** **PCR program used in this study**

| Number of Cycles | Time | Temperature | Step |
| --- | --- | --- | --- |
| 1 | 2 min | 95℃ | Initial denaturation |
| 35 | 2 min | 95℃ | Denaturation |
| 35 | 30 sec | 56 ℃ | Annealing |
| 35 | 1 min | 72 ℃ | Extension |
| 1 | 5 min | 72 ℃ | Final extension |
|  |  |  |  |
